# Hair Follicle-Related MicroRNA-34a Serum Expression and rs2666433A/G Variant in Patients with Alopecia: A Cross-Sectional Analysis

**DOI:** 10.3390/biom12050602

**Published:** 2022-04-19

**Authors:** Shymaa Ahmed Maher, Nader Ali Ismail, Eman A. Toraih, Alaa H. Habib, Nawal S. Gouda, Amal H. A. Gomaa, Manal S. Fawzy, Ghada M. Helal

**Affiliations:** 1 Department of Medical Biochemistry and Molecular Biology, Faculty of Medicine, Suez Canal University, Ismailia 41522, Egypt; shimaa.maher@med.suez.edu.eg; 2 Center of Excellence in Molecular and Cellular Medicine (CEMCM), Faculty of Medicine, Suez Canal University, Ismailia 41522, Egypt; 3 Department of Dermatology, Venereology and Andrology, Faculty of Medicine, Suez Canal University, Ismailia 41522, Egypt; naderali1967@hotmail.com (N.A.I.); ahhussein10@hotmail.com (A.H.A.G.); 4 Division of Endocrine and Oncologic Surgery, Department of Surgery, School of Medicine, Tulane University, New Orleans, LA 70112, USA; 5 Genetics Unit, Department of Histology and Cell Biology, Suez Canal University, Ismailia 41522, Egypt; 6 Department of Physiology, Faculty of Medicine, King Abdulaziz University, Jeddah 21589, Saudi Arabia; ahabib@kau.edu.sa; 7 Department of Medical Microbiology and Immunology, Faculty of Medicine, Mansoura University, Mansoura 35516, Egypt; nawalsalama@gmail.com; 8 Department of Biochemistry, Faculty of Medicine, Northern Border University, Arar 91431, Saudi Arabia; 9 Department of Medical Biochemistry, Faculty of Medicine, Mansoura University, Mansoura 35516, Egypt; ghadahelal76@mans.edu.eg

**Keywords:** alopecia areata, microRNA, miR-34a, gene expression, polymorphism, real-time PCR

## Abstract

Alopecia areata (AA) is a type of immune-mediated alopecia. Recent studies have suggested microRNAs’ (miRNAs) implication in several cellular processes, including epidermal and hair follicle biology. Single nucleotide polymorphisms (SNPs) can modify gene expression levels, which may induce an autoimmune response. This case–control study included 480 participants (240 for each case/control group). MicroRNA-34a gene (MIR-34A) rs2666433A/G variant was genotyped using real-time allelic discrimination polymerase chain reaction (PCR). Additionally, circulatory miR-34a levels were quantified by quantitative reverse transcription PCR (qRT-PCR). On comparing between alopecia and non-alopecia cohorts, a higher frequency of A variant was noted among patients when compared to controls—A allele: 28 versus 18% (*p* < 0.001); A/A genotype: 9 versus 2%; A/G genotype: 39 versus 32% (*p* < 0.001). A/A and A/G carriers were more likely to develop alopecia under heterozygote comparison (OR = 1.83, 95% CI = 1.14–2.93), homozygote comparison (OR = 4.19, 95% CI = 1.33–13.1), dominant (OR = 2.0, 95% CI = 1.27–3.15), recessive (OR = 3.36, 95% CI = 1.08–10.48), over-dominant (OR = 1.65, 95% CI = 1.04–32.63), and log additive (OR = 1.91, 95% CI = 1.3–2.82) models. Serum miR-34a expression levels were upregulated in alopecia patients with a median and quartile fold change of 27.3 (1.42–2430). Significantly higher levels were more pronounced in A/A genotype patients (*p* < 0.01). Patients carrying the heterozygote genotype (rs2666433 * A/G) were two times more likely to develop more severe disease grades. Stratified analysis by sex revealed the same results. A high expression level was associated with concomitant autoimmune comorbidities (*p* = 0.001), in particular SLE (*p* = 0.007) and vitiligo (*p* = 0.049). In conclusion, the MIR34A rs2666433 (A/G) variant is associated with AA risk and severity in the studied population. Furthermore, high miR-34a circulatory levels could play a role in disease pathogenesis.

## 1. Introduction

Widely spread in the human genome, microRNAs (miRNAs), small, highly conserved non-coding RNAs of about 19–26 nucleotides long, can regulate the expression of up to 60% of protein-coding genes [1]. Emerging studies have reported microRNAs as new key players in human diseases [2].

At present, the pathophysiological mechanisms of alopecia areata (AA), an autoimmune hair loss disease, are not completely clear, and the interplay between genetic predisposition, autoimmune compromise, and environmental factors is believed to contribute to disease development and progression [3,4,5]. Animal models have revealed discrete sets of differentially expressed miRNAs during epidermal morphogenesis [6]. They play essential roles in maintaining the highly proliferative matrix cells of the hair follicle in postnatal skin [7]. However, few studies have unraveled the role of miRNAs in the dynamic development and maturation of hair follicles in humans [8,9,10,11,12].

**Figure 1:**
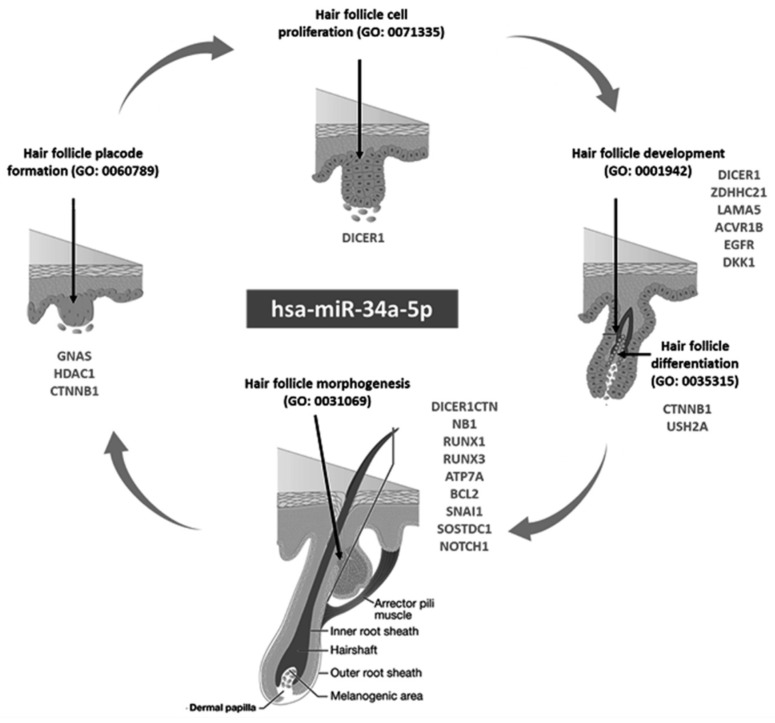
Functional enrichment analysis of miR-34a in hair follicle-related gene ontology terms. As depicted, miR-34a is involved in five biological processes. Significant, experimentally validated targeted genes in each pathway are listed [data source: http://diana.imis.athena-innovation.gr/] (accessed on 17 April 2021).

By exploring microRNA databases, hsa-miR-34a-5p was enriched in multiple hair follicle-related biological processes (Figure 1). It ranked as the first miRNA in hair follicle morphogenesis (GO: 0031069) via targeting twelve genes, including *DICER1*, *BCL2*, *NOTCH1*, *CTNNB1*, and *FGFR2*. The same miRNA was listed in the top miRNAs for hair cell differentiation (GO: 0035315), development (GO: 0001942), proliferation (GO: 0071335), and hair follicle placode formation (GO: 0060789). In addition, miR-34a-5p was the second most significant gene for the angiogenesis signaling pathway (GO: 0001525). These findings suggest that miR-34a plays an essential role in AA etiopathology and can be a potential candidate for individualized therapy in this disease.

**Figure 2:**
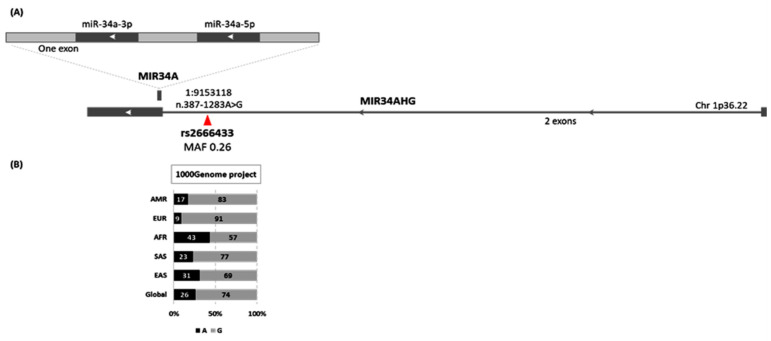
Genomic location (**A**) and population allele frequency of MIR34A rs2666433 polymorphism (**B**). AMR: America; EUR, Europe; AFR: Africa; SAS, South Asia; EAS: East Asia [Data source: enselmbl.org] (accessed on 17 April 2021).

The microRNA-34a gene (MIR34A) is located along the short arm of chromosome 1 (1p36.22), on the reverse strand within the second exon of the miR-34a-hosting gene (MIR34AHG). It encodes a single primary transcript, which produces both mature forms (miR-34a-5p and miR-34a-3p). Single nucleotide polymorphisms within the human genome could modulate microRNA regulation of gene silencing. Genetic variants in the regulatory region of miRNA genes might affect miRNA processing, maturation, and expression, while those in the miRNA-binding site might disrupt miRNA–mRNA interactions or create new targets [13]. Upstream to the MIR34A gene sequence, there is a common intronic variant within the MIRA34HG gene caused by the substitution of A to G (n.387-1283A/G) (Figure 2). This was recently associated with an increased risk of ischemic stroke [14].

Considering the key roles of microRNAs in inhibiting multiple gene signatures, deregulated expression of these biomolecules may also contribute to several disease development and/or severity, including AA [11]. In this sense, the authors were inspired to unravel the putative role of miR-34a-5p in AA by exploring the expression level of mature miR-34a-5p in serum of AA patients and genotyping its variant (rs2666433) for genotype–phenotype correlation. To the best of our knowledge, this is the first study investigating the association of this variant with AA susceptibility and severity.

## 2. Materials and Methods

### 2.1. Ethical Statement

This study was approved by the Ethics Committee of the College of Medicine, Suez Canal University (SCU), and was conducted according to the “Helsinki Declaration”. All recruited patients consented to participate in the study and publication of the results.

### 2.2. Study Participants

A total of 240 unrelated AA participants and 240 age- and sex-matched healthy controls were included in the study. Patients were recruited from the Department of Dermatology, Venerology, and Andrology, SCU Hospitals, Ismailia, and the Suez Canal area, Egypt. Patients who had spontaneous terminal hair regrowth received topical, intra-lesional, or systemic treatment within less than two weeks, or who had a history of persistent Alopecia Totalis/Universalis since infancy have been excluded. Additionally, patients with tumors or viral infections (e.g., hepatitis C/B or HIV) were excluded. However, patients with a history of other concomitant autoimmune diseases were included. Alopecia severity was categorized into mild, moderate, and severe according to the Kavak method [15]. Age and sex-matched volunteers were recruited from the Blood Bank of the SCU Hospital as healthy controls. They had no personal or family history of AA or other autoimmune diseases, including “inflammatory bowel disease, thyroid disease, systemic lupus erythematosus, psoriasis, or vitiligo”. A thorough history-taking and physical examination were done for control subjects before taking part to exclude incidental alopecia lesions.

In addition to the history taking and general/dermatological assessment, the “Severity of Alopecia Tool II (SALT)” score, advised by the National Alopecia Areata Foundation “NAAF” working committee [16], as described in our previous publications [4,5] was applied for each case. Poor prognostic indicators such as “young age at onset, positive family history of alopecia, atopy, severe phenotype, prolonged duration, nail disease, or associated autoimmune disease” were scored [17].

### 2.3. Blood Sampling and Genomic DNA Isolation

From all participants, venous blood samples were collected on EDTA (5 mL) and serum separator tubes (2 mL) under complete aseptic handling. Serum samples were kept in the refrigerator for at least 30 min and a maximum of 2 h between collection and centrifugation to allow the blood clotting. Then, the tubes were subjected to cold centrifugation for 20 min at 700× *g*, and the separated serum was aliquoted and stored at −80 °C until expression analysis. Genomic DNA was extracted from EDTA samples using a QIAamp DNA Blood Mini kit (Cat. No. 51104; Qiagen, Hilden, Germany) following the manufacturer’s guidelines. A NanoDrop ND-1000 (NanoDrop Technologies, Inc. Wilmington, DE, USA) was used to determine DNA concentration and purity.

### 2.4. MIR34A rs2666433A/G Genotyping

Real-time allelic discrimination polymerase chain reaction (PCR) was applied in a StepOne™ Real-Time PCR System (Applied Biosystems, Foster City, CA, USA) using a ready-made TaqMan assay (C___2800266_10, Cat. No. 4351379). The latter has the advantage of having “3′-minor groove binder-DNA probes” that enhance the sequence specificity at a wide range of PCR extension temperatures [18]. A final volume of 20 μL including 20 ng of the isolated DNA, 1 μL of the specified assay that contains sequence-specific forward and reverse primers, two allele-specific TaqMan minor groove-binding (MGB) probes that contain distinct fluorescent dyes [VIC/FAM] in the following specified context: [CCATGTGGGTGTGGGGGCATGAAGG[A/G]CTGTTTTCAGGAGGATCAAAGT CAC], respectively, and 10 μL of the Universal PCR Master Mix (Cat No. 4371353), was prepared for the PCR. An initial denaturation was executed for 10 min at 95 °C, followed by 40 cycles for 15 s at 90 °C and one minute at 60 °C. Non-template (NTC) and no-enzyme negative controls were run with the samples to ensure contamination-free runs. Ten percent of the samples were run in duplicate, with a 100% concordance rate for genotype calls.

### 2.5. MIR34A Expression Profiling

The expression of mature miR-34a (i.e., hsa-miR-34a-5p) was profiled in 114 cases and 86 controls. The total RNA, including small RNA, was extracted from the archived serum using a “Qiagen miRNeasy Serum/Plasma Kit (Catalog no. 217184, Qiagen)”, following the manufacturer’s guidelines. Then, RNA quality and purity were assessed as mentioned above. Reverse transcription (RT) was run using miRNA-specific primer 5x (for the Stem-loop Sequence “GGCCAGCUGUGAGUGUUUCUUUGGCAGUGUCUUA GCUGGUUGUUGUGAGCAAUAGUAAGGAAGCAAUCAGCAGUAUACUGCCCUAGAAGUGCUGCACGUUGUGGGGCCC” and the RT (P/N 4366596, ThermoFisher, Waltham, MA, USA) [19,20]. Amplification of the complementary DNA in the RT reactions was done in the “Mastercycler Gradient Thermocycler (Eppendorf, Hamburg, Germany)”. Mature hsa-miR-34a-5p was quantified by the Real-Time PCR protocol using TaqMan MicroRNA assay (20×) (assay ID 000426, ThermoFisher, Waltham, MA, USA) for the mature miRNA Sequence: “UGGCAGUGUCUUAGCUGGUUGU” according to the Build GRCh38 (https://www.thermofisher.com/order/genome-database/details/mirna/000426, accessed on 10 March 2022), the internal control RNU6B primer set (assay ID:001093, cat. no 4427975, ThermoFisher Scientific, Waltham, MA, USA) which is specific for the “CGCAAGGATGACACGCAAATTCGTGAAGCGTTCCATA TTTTT” sequence (https://www.thermofisher.com/order/genome-database/details/microrna/001093) (accessed on 13 March 2022), and TaqMan Universal PCR Master Mix II (2×) (P/N 4440043), in a final volume of 20 µL, including “1.33 µL RT products, 2 × TaqMan Universal Master Mix II, and 1 µL TaqMan MicroRNA assay/RNU6B assay”. The PCR program included initial denaturation for 10 min at 95 °C, followed by 40 cycles for 15 s at 92 °C, and one minute at 60 °C. NTC samples were also included in each run. The “minimum information for the publication of quantitative real-time PCR experiments (MIQE)” guideline was followed [21]. The fold change of MIR34A gene expression was calculated according to the following equation: = 2^−ΔΔCq^, where delta delta quantitative cycle (ΔΔC_q_) = (C_q_ MIR34A-C_q_ RNU6B) _alopecia patients_ − (C_q_ MIR34A-C_q_ RNU6B) _control_ _group_ [22].

### 2.6. Statistical Analysis

General statistical analyses were performed with Statistical Package for Social Science (SPSS) software version 26 (IBM Corp, Armonk, NY, USA). The Kolmogorov–Smirnov test was run to test the normality of quantitative data presented as mean ± standard deviation (SD) or median (quartiles). The allele/genotype frequencies were compared between the patients and controls using the Chi-squared test as previously described [23]. Estimation of Hardy–Weinberg equilibrium (HWE) was done by the online tool (http://www.oege.org/software/hwe-mr-calc.shtml, accessed on 6 September 2021). SNP analysis was conducted using the SNPstats web tool (www.snpstats.net, accessed on 6 September 2021). Odds ratios (OR) with a 95% confidence interval (CI) were calculated for each genetic association model [23]. Statistical differences between groups regarding their clinical features were tested using the Chi-squared, Fisher’s Exact, Student’s *t*-, and Mann–Whitney U tests according to data type and distribution. The Spearman’s test was applied to test the fold change of MIR34A expression correlation with the different genotypes. A two-tailed *p*-value of < 0.05 was considered statistically significant.

## 3. Results

### 3.1. Characteristics of the Study Population

**Figure 3:**
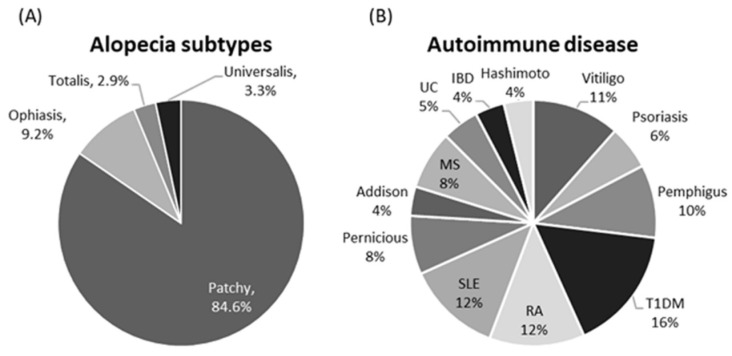
Types of alopecia phenotypes (**A**) and concomitant autoimmune diseases (**B**) in alopecia patients. Data presented as frequency (percentage).

The demographic features of the studied participants are shown in Table 1. There was no significant difference between the two studied groups. Most of the patients had patchy scalp lesions (203 patients, 84.6%), while alopecia totalis and universalis accounted for 2.9 and 3.3% of cases, respectively, who exhibited poor prognostic index (*p* = 0.027) and high SALT score (*p* < 0.001) (Table 2). Diabetes type 1, SLE, and RA were the most prevalent concomitant autoimmune diseases (Figure 3).

### 3.2. Genotyping of MIR34A Polymorphism

**Figure 4:**
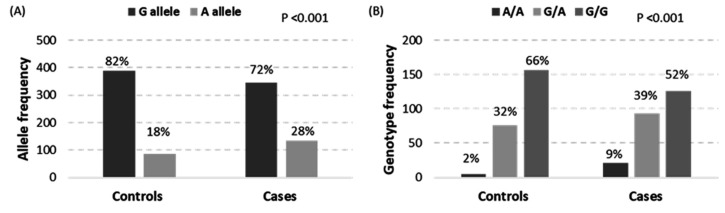
Allele (**A**) and genotype (**B**) frequencies of MIR34A rs2666433 polymorphism. Counts are represented along the y axis, and the percentage is shown over the bar. A Chi-squared test was used. *p*-value < 0.05 was considered as statistically significant.

Genotyping of 240 patients/240 controls were in agreement with HWE (*p* = 0.52 and 0.28, respectively). Minor allele frequency (MAF; A allele) represented 23% of the total sample. Correspondingly, A/A and A/G genotypes accounted for 5 and 35%, respectively, in the study population. On comparing between alopecia and non-alopecia cohorts, a higher frequency of A variant was noted among patients when compared to controls (A allele: 28% versus 18% (*p* < 0.001), A/A genotype: 9% versus 2%, and A/G genotype: 39% versus 32% (*p* < 0.001)) (Figure 4).

### 3.3. Impact of rs2666433 Genotype on Disease Risk Transcriptomic Signature of miR-34a

Disease risk assessment by six genetic association models showed that A/A and A/G carriers were more likely to develop alopecia under heterozygote comparison (OR = 1.83, 95% CI = 1.14–2.93), homozygote comparison (OR = 4.19, 95% CI = 1.33–13.1), dominant model (OR = 2.0, 95% CI = 1.27–3.15), recessive model (OR = 3.36, 95% CI = 1.08–10.48), over-dominant model (OR = 1.65, 95% CI = 1.04–32.63), and log additive model (OR = 1.91, 95% CI = 1.3–2.82) (Table 3). In the study, males represented 187 controls and 191 patients, while females accounted for 45 controls and 28 cases. Stratification by sex revealed inconsistent findings, with only males having high risk (Supplementary Table S1).

### 3.4. Transcriptomic Signature of miR-34a

**Figure 5:**
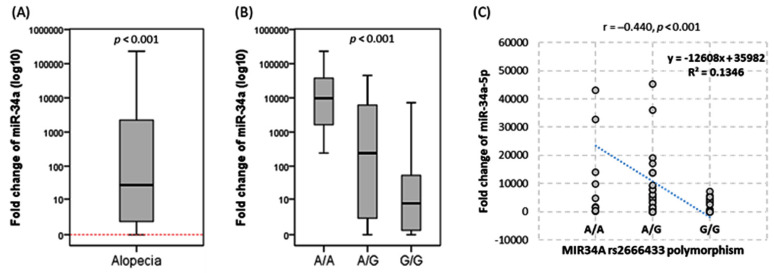
Fold change of miR-34a-5p in alopecia cases and controls. (**A**) Gene up-regulation in alopecia versus controls (red dotted line). The Mann-Whitney U test was used. (**B**) Expression level according to the MIR34A gene polymorphism. The Kruskal–Wallis test was employed. (**C**) Correlation analysis between expression and genotypes. The Spearman’s correlation test was used. The correlation coefficient (r), R2, and regression equation are shown. Statistical significance was set at a *p*-value < 0.05.

Marked upregulation of miR-34 was observed in the circulation of alopecia patients with median and quartile fold change of 27.3 (1.42–2430). Significantly higher levels were more pronounced in A/A genotype patients (*p* < 0.01) (Figure 5).

### 3.5. Association of mir-34a Expression and Variant with Disease Severity

Patients were classified according to severity in Table 4. Patients carrying the heterozygote genotype (rs2666433 * A/G) were two times more likely to develop more severe disease grade under heterozygote comparison (A/G versus G/G: OR = 2.33, 95% CI = 1.29–4.22), dominant model (A/G-A/A versus G/G: OR =2.18, 95% CI = 1.25–3.8), and over-dominant model (A/G versus both A/A-G/G: OR = 2.16, 95% CI = 1.21–3.84). Stratified analysis by sex revealed the same results; heterozygosity was associated with higher susceptibility of advanced severity in males (OR = 2.04, 95% CI = 1.09–3.83) and females (OR = 6.34, 95% CI = 1.04–38.49). High expression level was associated with concomitant autoimmune comorbidities (*p* = 0.001), in particular, SLE (*p* = 0.007) and vitiligo (*p* = 0.049) (Table 5).

## 4. Discussion

Alopecia areata is one of the most frequent types of hair loss in humans. Its etiopathology exhibits substantial heterogeneity, comprising a complex interplay of immunological, hormonal, environmental, inflammatory, apoptotic, oxidative stress, genetic, and epigenetic variables [24]. Discovering the genetic/epigenetic component behind alopecia pathogenesis will pave the road for better diagnosis, treatment, and prognosis [25]. The microRNA family of non-coding RNAs has been related to immune system homeostasis, and genetic variants and gene signature dysregulation have been connected to several immunological diseases, including alopecia [26,27].

To the best of our knowledge, this is the first study to investigate the relationship between the MIR34A rs2666433 variant and gene expression in alopecia. The current study’s key findings are (I) MIR34A rs2666433 A/A and A/G genotypes are associated with the risk of alopecia in the study population; (II) heterozygote genotype rs2666433 * A/G was twice as likely to develop more severe disease grade; (III) serum miR-34a expression levels were elevated in alopecia patients when compared to controls. Our findings suggest that the rs2666433 polymorphism and miR-34a may play a role in alopecia susceptibility.

Previous research by Wei et al. has shown that in the Chinese population, the rs2666433 variant was associated with a significantly increased risk of ischemic stroke, particularly in the large artery atherosclerosis subtype [14]. These findings are consistent with ours regarding the association of the A/A genotype with higher transcript levels than carriers of the G/G and G/A genotypes, which may open up new avenues that this variation may influence gene expression levels. The rs2666433 AA/AG genotype has been associated with colorectal cancer risk, and a high somatic mutation rate in cancer tissues, as well as gene overexpression, was evident in the tissue specimens of this type of cancer [28]. Furthermore, miR-34a was over-expressed in both T2DM and diabetic nephropathy patients, and rs2666433 A/G in miR-34a is associated with an increased risk of T2DM; this raises the possibility that this variation may affect the binding of transcription factors to this gene promoter region [29]. On the other hand, Ismail and colleagues revealed that the MIR34A rs2666433 variant conferred protection against developing SLE disease in their study population and showed no association with disease activity [30].

An in silico analysis for rs2666433 SNP using the online tool “HaploReg V4.1” for expression quantitative trait locus (eQTL) analysis [30] revealed that our studied rs2666433 SNP was in linkage disequilibrium (r^2^ = 0.8) with other variants such as rs113390912, rs34174278, rs34196792, and rs34619897 that are present on chromosome 1. It can influence and alter the peroxisome proliferator-activated receptor (PPAR), Erythroblast Transformation-specific (ETs), and the paired box-4 (Pax-4) DNA motifs that were shown to be linked to alopecia [31,32,33,34]. Thus, we speculated that alteration of these motifs might contribute to the association between rs2666433 and the development of alopecia.

The motifs mentioned above have been shown to play a role in normal physiological skin and appendage-related processes, and their alterations have been reported to be associated with skin disease-related conditions. For example, (1) PPARγ is a member of the PPAR nuclear hormone receptors, which are abundantly expressed in tissues with high fatty acid metabolisms, such as human skin and appendages, especially HFs [33]. The PPAR-mediated signaling has been linked to psoriasis, atopic dermatitis, acne, skin aging, scleroderma, melasma, lipodystrophy, and skin cancer [31,35,36]. It is implicated in the signaling pathway that suppresses epithelial-to-mesenchymal transition (EMT), which plays a role in lichen planaris, frontal fibrosing alopecia, and chemotherapy-induced alopecia [33,34,37,38]. PPAR-γ also plays a significant role in HF homeostasis, as its stimulation results in increased mitochondrial energy metabolism, premature catagen development, and suppression of intra- and perifollicular inflammation in hair bulge, in HFs affected by lichen planopilaris [32,34,39]. Furthermore, in vivo deletion of PPAR-γ in mice results in an LPP-like phenotype with progressive hair loss, perifollicular inflammation, and scarring alopecia [38]. (2) The transcriptional factor (ETs) is involved in a wide range of cellular processes, including cell differentiation, cell cycle control, migration, proliferation, apoptosis, and angiogenesis [40]. Some members of this family, such as ETV2, have been found to promote hair growth through vascular regeneration in chemotherapy-induced alopecia [41], while Elf5, another ET family member, is expressed in the inner root sheath of hair follicles [42]. The functional participation of these target genes may explain the current study’s significant association between the investigated polymorphism and the development of alopecia and other phenotypic traits. Future functional studies are recommended to confirm these speculated associations.

Deregulation of miRNA has been implicated in the pathophysiology of common hair loss disorders such as AA, androgenetic alopecia, and frontal fibrosing alopecia [27,43,44,45]. It was reported that patients with AA presented with abnormal levels of miRNAs in the epidermis and T lymphocyte cells attacking the follicle when compared to healthy participants [46,47,48]. For example, miR-210 and miR-1246 were upregulated in AA and associated with the active phase of the disease, and miR-30b may contribute to AA development [49,50]. In line with these previous findings, we also found a significant upregulation of circulating miR-34a in our patients with AA when compared to controls.

MiR-34a has been related to the pathogenesis of skin diseases [51,52,53] but has not been studied before in alopecia. In the current study, in silico functional enrichment analysis confirmed the implication of miR-34a-5p in five biological processes by targeting several genes related to hair follicle development, morphogenesis, proliferation differentiation, and hair follicle placode formation. For example, one of the mir-34a target genes is *DICER1* (Figure 1), a miRNA-processing enzyme that has been proven to play a crucial role in skin and HF biology. It was reported that loss of epithelial cells’ Dicer causes defective morphogenesis and reduced hair follicle proliferation and epidermal differentiation through an effect on signaling pathways involved in such process as sonic hedgehog(shh), and NOTCH 1, as well as causing loss of hair-follicle stem cell marker expression, which results in a loss of differentiation and degeneration [54].

Additionally, mir-34a is considered one of the mitomiRs (mitochondria-related microRNAs) that suppress the antioxidant and antiapoptotic mitochondrial protein expression of Bcl-2, which leads to an increase in reactive oxygen species production, permeability transition pore opening, and the activation of caspase-1 and -3. Thus, its activity can contribute to mitochondrial dysfunction, increased oxidative stress, chronic inflammation, and increased cell death [55], which may contribute to AA pathogenesis [56].

Another downstream target of mir-34a is *LAMA5* (Figure 1). Using microarray analysis, Lim and colleagues identified this target involved in the biological regulation of the hair cycle, hair follicle development, maturation, and morphogenesis [57]. Another downstream target is Histone deacetylases 1 (*HDAC1*), a zinc-dependent enzyme that removes the acetyl group from histones and other proteins, allowing for epigenetic regulation of gene expression. In addition to its immunomodulatory functions, it plays a vital role in the hair follicle and epidermal homeostasis. HDAC1 appears to be dysregulated in AA and acne vulgaris individuals [58].

Interestingly, the current study discovered that miR-34a expression was associated with concomitant autoimmune comorbidity, SLE, and vitiligo. Previously, it was reported that deregulated expression of miR-34a was observed in several autoimmune disorders, including SLE, and in lupus nephritis, miR-34a expression in renal tissues was higher in LN-SLE patients when compared to IgA nephropathy patients [59]. In rheumatoid arthritis (RA), inflammatory cytokines bind to the miR34a promoter, increasing its production. Sustained miR-34a expression in CD4+ T cells from RA patients or arthritis mouse models influences cellular Foxp3 expression, altering Treg cell polarization/generation and aggravating RA progression [60]. A recent intriguing study regarding relationships between miRNAs and genes encoding transcription factors, ubiquitylation, DNA methylation, and histone modifications in SLE revealed that altered hsa-miR-34a-5p was among the most frequent miRNAs associated with susceptibility-related genes [61].

Additionally, overexpression of miR-34a was reported in Crohn’s disease and found to exert a pro-fibrotic effect [38]. Interestingly, alopecia areata has been considered one of the extraintestinal cutaneous manifestations of Crohn’s disease. Given that alopecia areata and Crohn’s disease are considered inflammatory Th1-mediated diseases [62], it is not surprising that dysregulation of miRNA expression could be found in multiple autoimmune diseases that share similarities with the immune-mediated etiopathology.

Endoplasmic reticulum stress and unfolded protein response (UPR) have been shown to play a role in skin diseases. It was reported that miR-34a-5p overexpression influences UPR via a substantial reduction in IRE1 and XBP1s, which leads to decreased viability, increased cytotoxicity, and caspase activity [63,64]. It has also been observed that miR-34a-5p inhibits SIRT1, which has been shown to reduce oxidative stress, reducing ROS accumulation, and apoptosis [65,66]. Mir34a downregulation of SIRT1 promotes apoptosis and activates the IL-1β/COX2/PGE2 inflammatory pathway [67]. Human scalp areas affected by alopecia areata show an excessive expression of IL-1β at the early stages of the disease [68]. Furthermore, SIRT1 was implicated in the pathogenesis of vitiligo and contributes to skin cell damage via regulating several downstream signaling pathways [69,70].

MiR-34a has also been proven to target the vascular endothelial growth factor (VEGF), an essential player in angiogenesis and vascular permeability, supporting a healthy vasculature surrounding the hair follicles [71]. Reduced VEGF production by hair follicles has been seen in AA, suggesting a significant contributing role in hair loss diseases [72]. Reduced VEGF production by hair follicles may reduce vascular support to affected scalp tissue, leading to inflammatory changes. Additionally, VEGF has been shown to have antiapoptotic properties during angiogenesis, mediated by the induced expression of survivin [73]; this suggests a potential contributing mechanism through which mir-34a overexpression could affect AA pathogenesis.

Interestingly, when genotyping data were stratified by sex, the rs2666433 variant showed more frequency in male participants than females, with males having a higher risk, although the number of males in this study was nearly four times that of females, even though this does not negate thoughts about the existence of a possible relation between androgen and miR-34a. Androgens are thought to derive pathophysiological cascades in genetically sensitive scalp dermal papilla, eventually culminating in patterned alopecia. Several microRNAs have been reported to be upregulated and to contribute to the pathophysiology of androgenic alopecia, including miR-221, miR-125b, miR-106b, miR-410, and miR-133b [26,74]. The androgen receptors (AR)–miR-34a interaction has been demonstrated previously in renal cell carcinoma and prostate cancer [75,76].

## 5. Conclusions

The current investigation unraveled an association between MIR34A expression and its variant (rs266643) A/A and A/G genotypes with susceptibility to alopecia areata, supporting the potential miR-34a involvement in the pathogenesis of AA and the possible utility of this biomolecule as a potential molecular marker for AA. Further large-scale research with functional studies is recommended to confirm this conclusion.

## Figures and Tables

**Table 1:** Demographic characteristics of the study population.

Variables	Categories	Controls	AA	*p*-Value	OR (95% CI)
Age, years	Mean ± SD	29.3 ± 5.4	30.7 ± 7.1	0.09	
	≤30 years	142 (59.2)	120 (50)	0.05	
	>30 years	98 (40.8)	120 (50)		1.44 (0.99–2.07)
Sex	Male	194 (80.8)	208 (86.7)	0.10	
	Female	46 (19.2)	32 (13.3)		0.64 (0.39–1.06)
BMI, Kg/m^2^	Mean ± SD	25.4 ± 2.6	25.8 ± 2.8	0.08	
Obesity	Negative	230 (95.8)	222 (92.5)	0.17	
	Positive	10 (4.2)	18 (7.5)		1.85 (0.84–4.12)
Residence	Port-said	18 (7.5)	13 (5.4)	0.22	
	Suez	21 (8.8)	15 (6.3)		
	Ismailia	59 (24.6)	77 (32.1)		
	Cairo	142 (59.2)	135 (56.3)		
Occupation	Student	96 (40)	106 (44.2)	0.17	
	Unemployed	97 (40.4)	102 (42.5)		
	Employed	47 (19.6)	32 (13.3)		
Family history	Alopecia	0 (0)	106 (44.2)	NA	
	Autoimmune dis	0 (0)	103 (42.9)	NA	

Data are shown as number (percentage) or mean ± SD. The Chi-squared test was used for qualitative variables, and the Student’s *t*-test was used for quantitative variables. *p*-value < 0.05 was considered as statistically significant. OR (95% CI), odds ratio, and confidence interval.

**Table 2:** Clinical characteristics of alopecia patients.

Characteristics		Patchy and Aphiasis	Totalis and Universalis	*p*-Value	OR (95% CI)
Number		225	15		
Demographic characteristics					
Age, year	≤30 years	113 (50.2)	7 (46.7)	0.37	1.50 (0.67–3.33)
	>30 years	112 (49.8)	8 (53.3)		
Sex	Male	195 (86.7)	13 (86.7)	1.00	1.00 (0.98–1.10)
	Female	30 (13.3)	2 (13.3)		
Obesity	Negative	207 (92)	15 (100)	0.61	0.85 (0.63–1.16)
	Positive	18 (8)	0 (0)		
Residence	Port-said	12 (5.3)	1 (6.7)	0.68	
	Suez	13 (5.8)	2 (13.3)		
	Ismailia	73 (32.4)	4 (26.7)		
	Cairo	127 (56.4)	8 (53.3)		
Occupation	Student	97 (43.1)	9 (60)	0.82	
	Unemployed	96 (42.7)	6 (40)		
	Employed	32 (14.2)	0 (0)		
Family history of alopecia	Negative	126 (56)	8 (53.3)	0.53	0.83 (0.58–1.19)
	Positive	99 (44)	7 (46.7)		
Family history of autoimmune disease	Negative	129 (57.3)	8 (53.3)	0.79	0.83 (0.53–1.19)
	Positive	96 (42.7)	7 (46.7)		
Prior episode of alopecia	Negative	73 (32.4)	8 (53.3)	0.15	0.42 (0.87–1.02)
	Positive	152 (67.6)	7 (46.7)		
Duration of disease, month	≤1 years	190 (84.4)	15 (100)	0.40	0.92 (0.89–0.96)
	>1 years	35 (15.6)	0 (0)		
Age at onset	≤20 years	205 (91.1)	12 (80)	0.163	2.56 (0.66–9.84)
	>20 years	20 (8.9)	3 (20)		
Disease characteristics					
Nail changes	Negative	145 (64.4)	12 (80)	0.27	0.83 (0.58–1.19)
	Positive	80 (35.6)	3 (20)		
Itching	Negative	169 (75.1)	15 (100)	0.40	0.80 (0.51–1.24)
	Positive	56 (24.9)	0 (0)		
Scalp infection	Negative	164 (72.9)	10 (66.7)	0.56	1.34 (0.44–4.09)
	Positive	61 (27.1)	5 (33.3)		
Concomitant comorbidity	None	154 (68.4)	12 (80)	0.64	
	Single	47 (20.9)	2 (13.3)		
	Multiple	24 (10.7)	1 (6.7)		
Hypertension	Negative	205 (91.1)	14 (93.3)	0.76	0.73 (0.09–5.86)
	Positive	20 (8.9)	1 (6.7)		
Atopy	Negative	171 (76)	12 (80)	0.50	0.79 (0.21–2.91)
	Positive	54 (24)	3 (20)		
Emotional stress	Negative	59 (26.2)	1 (6.7)	0.12	4.9 (0.64–38.6)
	Positive	166 (73.8)	14 (93.3)		
SALT score, %	Median (quartiles)	8 (6–11)	100	**<0.001**	
Prognostic index	Mean ± SD	1.69 ± 1.17	2.26 ± 0.88	**0.027**	
DLQI score	Mean ± SD	13.1 ± 10.0	12.4 ± 9.8	0.52	
Responded to treatment	Negative	63 (28)	1 (6.7)	0.07	5.44 (0.70–4.2)
	Positive	162 (72)	14 (93.3)		

Data are shown as number (percentage), mean ± SD, or median (quartiles). Chi-squared and Fisher’s Exact tests were used for categorical variables, and the Student’s t- and Mann-Whitney U tests were applied for quantitative variables. Bold *p*-values < 0.05 were considered statistically significant. OR (95% CI), odds ratio and confidence interval between (alopecia totalis and universalis versus patchy and aphiasis alopecia areata). BMI: body mass index; SALT: Severity of Alopecia Tool score for severity assessment; DLQI: Dermatology Life Quality Index questionnaire.

**Table 3:** Risk of developing alopecia disease by genetic association models of MIR34A rs2666433 genotype.

Model	Genotype	Controls	Cases	Crude OR (95% CI)	*p*-Value	Adjusted OR (95% CI)	*p*-Value
Codominant	G/G	156 (65.8%)	126 (52.5%)	1.00	**4 × 10^−4^**	1.00	**0.0041**
	A/G	76 (32.1%)	93 (38.8%)	1.52 (1.03–2.22)		1.83 (1.14–2.93)	
	A/A	5 (2.1%)	21 (8.8%)	5.20 (1.91–14.18)		4.19 (1.33–13.19)	
Dominant	G/G	156 (65.8%)	126 (52.5%)	1.00	**0.003**	1.00	**0.0028**
	A/G-A/A	81 (34.2%)	114 (47.5%)	1.74 (1.20–2.52)		2.00 (1.27–3.15)	
Recessive	G/G-A/G	232 (97.9%)	219 (91.2%)	1.00	**9 × 10^−4^**	1.00	**0.03**
	A/A	5 (2.1%)	21 (8.8%)	4.45 (1.65–12.00)		3.36 (1.08–10.48)	
Overdominant	G/G-A/A	161 (67.9%)	147 (61.2%)	1.00	0.13	1.00	**0.033**
	A/G	76 (32.1%)	93 (38.8%)	1.34 (0.92–1.95)		1.65 (1.04–2.63)	
Log-additive	-	-	-	1.77 (1.30–2.43)	**2 × 10^−4^**	1.91 (1.30–2.82)	**0.001**

Values are shown as numbers (%). A Chi-squared test was used. OR (95% CI), odds ratio, and confidence interval. Bold *p*-values < 0.05 were considered statistically significant. Adjusted covariates: age, sex, BMI, occupation, residency, and family history. Codominant models included both heterozygote comparison (A/G versus G/G) and homozygote comparison (A/A versus G/G).

**Table 4:** Risk of severe phenotype in alopecia patients with MIR34A rs2666433.

Model	Genotype	Mild	Moderate/Severe	Crude OR (95% CI)	*p*-Value	Adjusted OR (95% CI)	*p*-Value
Codominant	G/G	72 (62.6%)	54 (43.2%)	1.00	**0.009**	1.00	**0.017**
	A/G	34 (29.6%)	59 (47.2%)	2.31 (1.33–4.01)		2.33 (1.29–4.22)	
	A/A	9 (7.8%)	12 (9.6%)	1.78 (0.70–4.52)		1.70 (0.64–4.50)	
Dominant	G/G	72 (62.6%)	54 (43.2%)	1.00	**0.002**	1.00	**0.005**
	A/G-A/A	43 (37.4%)	71 (56.8%)	2.20 (1.31–3.69)		2.18 (1.25–3.80)	
Recessive	G/G-A/G	106 (92.2%)	113 (90.4%)	1.00	0.63	1.00	0.69
	A/A	9 (7.8%)	12 (9.6%)	1.25 (0.51–3.09)		1.21 (0.47–3.12)	
Overdominant	G/G-A/A	81 (70.4%)	66 (52.8%)	1.00	**0.004**	1.00	**0.008**
	A/G	34 (29.6%)	59 (47.2%)	2.13 (1.25–3.63)		2.16 (1.21–3.84)	
Log-additive	-	-	-	1.68 (1.12–2.52)	**0.011**	1.64 (1.07–2.51)	**0.021**

Values are shown as numbers (%). A Chi-squared test was used. OR (95% CI), odds ratio, and confidence interval. Bold *p*-values < 0.05 were considered statistically significant. Adjusted covariates: age, sex, BMI, occupation, residency, and family history.

**Table 5:** Univariate association analysis of miR-34a expression and variant with demographic/clinical features of alopecia patients.

Characteristics		No. of Cases	Fold Change	*p*-Value	Genotype			*p*-Value
			Median (Quartiles)		AA	AG	GG	
Age	≤30 years	120 (50)	35.2 (1.8–4238.7)	0.26	10 (47.6)	50 (53.8)	60 (47.6)	0.65
	>30 years	120 (50)	14.1 (0.4–983.4)		11 (52.4)	43 (46.2)	66 (52.4)	
Sex	Male	208 (86.7)	20.1 (1.5–1698.5)	0.33	17 (81)	83 (89.2)	108 (85.7)	0.54
	Female	32 (13.3)	877.4 (0.9–5442)		4 (19)	10 (10.8)	18 (14.3)	
Obesity	Negative	222 (92.5)	17.2 (1.3–2687.7)	0.81	20 (95.2)	90 (96.8)	112 (88.9)	0.08
	Positive	18 (7.5)	161.2 (2.1–249.1)		1 (4.8)	3 (3.2)	14 (11.1)	
Family history of alopecia	Negative	134 (55.8)	30.1 (1.6–3501.9)	0.33	10 (47.6)	60 (64.5)	64 (50.8)	0.09
	Positive	106 (44.2)	13.3 (0.4–1067.1)		11 (52.4)	33 (35.5)	62 (49.2)	
Family history autoimmune dis	Negative	137 (57.1)	29.6 (2–3400.3)	0.34	10 (47.6)	60 (64.5)	67 (53.2)	0.16
	Positive	103 (42.9)	25.2 (0.4–457.2)		11 (52.4)	33 (35.5)	59 (46.8)	
Age at onset	≤20 years	217 (90.4)	29.6 (1.5–2601.5)	0.83	20 (95.2)	82 (88.2)	115 (91.3)	0.54
	>20 years	23 (9.6)	17.2 (0.9–2540.4)		1 (4.8)	11 (11.8)	11 (8.7)	
Duration of disease	≤12 months	205 (85.4)	29.6 (1.5–2601.5)	0.22	19 (90.5)	78 (83.9)	108 (85.7)	0.73
	>12 months	35 (14.6)	17.2 (0.9–2540.4)		2 (9.5)	15 (16.1)	18 (14.3)	
Prior episode of alopecia	Negative	81 (33.8)	10.4 (1.2–1480.7)	0.86	7 (33.3)	33 (35.5)	41 (32.5)	0.90
	Positive	159 (66.3)	34.9 (1.6–2687.7)		14 (66.7)	60 (64.5)	85 (67.5)	
Nail changes	Negative	157 (65.4)	11.5 (0.7–1703.3)	0.25	13 (61.9)	67 (72)	77 (61.1)	0.22
	Positive	83 (34.6)	51.5 (2–3677.2)		8 (38.1)	26 (28)	49 (38.9)	
Itching	Negative	184 (76.7)	29.6 (1.3–2882.4)	0.93	16 (76.2)	77 (82.8)	91 (72.2)	0.09
	Positive	56 (23.3)	25.2 (1.9–504.2)		5 (23.8)	16 (17.2)	35 (27.8)	
Scalp infection	Negative	174 (72.5)	14.9 (0.7–2601.5)	0.57	13 (61.9)	65 (69.9)	96 (76.2)	0.30
	Positive	66 (27.5)	29.6 (2.1–2302.6)		8 (38.1)	28 (30.1)	30 (23.8)	
Atopy	Negative	183 (76.3)	14.6 (0.4–2451.1)	0.16	16 (76.2)	72 (77.4)	95 (75.4)	0.94
	Positive	57 (23.8)	51.5 (3.3–2601.5)		5 (23.8)	21 (22.6)	31 (24.6)	
Hypertension	Negative	219 (91.3)	14.9 (0.9–1640.1)	0.05	21 (100)	82 (88.2)	116 (92.1)	0.20
	Positive	21 (8.8)	249.1 (7.7–7219.4)		0 (0)	11 (11.8)	10 (7.9)	
Emotional stress	Negative	60 (25)	14.1 (0.6–1783.6)	0.74	6 (28.6)	17 (18.3)	37 (29.4)	0.16
	Positive	180 (75)	29.6 (1.6–2806.6)		15 (71.4)	76 (81.7)	89 (70.6)	
Concomitant autoimmune disease	Negative	166 (69.2)	3.7 (0.3–1384.3)	**0.001**	14 (66.7)	63 (67.7)	89 (70.6)	0.87
	Positive	74 (30.8)	64.7 (6.6–4804.9)		7 (33.3)	30 (32.3)	37 (29.4)	
T1DM	Negative	223 (92.9)	30.6 (1.3–1917.5)	0.92	20 (95.2)	89 (95.7)	114 (90.5)	0.30
	Positive	17 (7.1)	10.4 (2.1–3897.3)		1 (4.8)	4 (4.3)	12 (9.5)	
SLE	Negative	227 (94.6)	14.6 (1.1–1480.7)	**0.007**	21 (100)	84 (90.3)	122 (96.8)	0.05
	Positive	13 (5.4)	4191.1 (179.9–13704)		0 (0)	9 (9.7)	4 (3.2)	
RA	Negative	227 (94.6)	29.6 (1.2–2925.5)	0.48	21 (100)	90 (96.8)	116 (92.1)	0.16
	Positive	13 (5.4)	3.4 (1.9–48.3)		0 (0)	3 (3.2)	10 (7.9)	
Vitiligo	Negative	228 (95)	14.6 (1.1–1739)	**0.049**	20 (95.2)	85 (91.4)	123 (97.6)	0.11
	Positive	12 (5)	887.7 (47.8–8190)		1 (4.8)	8 (8.6)	3 (2.4)	
Responded to treatment	Negative	64 (26.7)	13.3 (0.4–887.3)	0.15	3 (14.3)	23 (24.7)	38 (30.2)	0.27
	Positive	176 (73.3)	34.9 (1.7–3197.1)		18 (85.7)	70 (75.3)	88 (69.8)	

Expression level is shown as median (quartiles) and frequency as number (percentage). Chi-squared and Fisher’s Exact tests were used for categorical variables, and Mann-Whitney U tests were applied for quantitative variables. Bold *p*-values < 0.05 were considered statistically significant. T1DM: type 1 diabetes mellitus; SLE: systemic lupus erythematosus; RA: rheumatoid arthritis.

## Data Availability

All generated data in this study are included in the article.

## Supplementary Materials

The following supporting information can be downloaded at: https://www.mdpi.com/article/10.3390/biom12050602/s1, Table S1: Stratified analysis by sex for genotype frequencies between cases.
