# Supplementary material for: Hair Follicle-Related MicroRNA-34a Serum Expression and rs2666433A/G Variant in Patients with Alopecia: A Cross-Sectional Analysis
Source: Biomolecules. 2022 Apr 19;12(5):602. doi: 10.3390/biom12050602 (PMC9138785; doi:10.3390/biom12050602)
Supplement: Supplementary file 1 [file biomolecules-12-00602-s001.zip › biomolecules-1659210-supplementary.pdf]

Supplementary Table S1. Stratified analysis by sex for genotype frequencies between cases and controls

	**Males**				**Females**				
	**Controls**	**Cases**	**Crude OR (95% CI)**	**Adjusted OR (95% CI)**	**Controls**	**Cases**	**OR (95% CI)**	**Adjusted OR (95% CI)**	***P* interaction**
G/G	125	108	1.00	1.00	31	18	1.00	1.00	0.24
A/G	62	83	**1.55 (1.02–2.35)**	**1.81 (1.08–3.02)**	14	10	1.23 (0.45–3.34)	1.88 (0.59–6.00)	
A/A	5	17	**3.94 (1.41–11.02)**	**3.67 (1.14–11.82)**	0	4	---	---	

Values are shown as numbers (%). A Chi-square test was used. OR (95% CI), odds ratio, and confidence interval. Bold *p*-values < 0.05 were considered statistically significant. Adjusted covariates: age, sex, BMI, occupation, residency, and family history.
